# Orofacial clefts alter early life oral microbiome maturation towards higher levels of potentially pathogenic species: A prospective observational study

**DOI:** 10.1080/20002297.2022.2164147

**Published:** 2023-01-04

**Authors:** Corinna L. Seidel, Karin Strobel, Matthias Weider, Marco Tschaftari, Christoph Unertl, Ines Willershausen, Manuel Weber, André Hoerning, Patrick Morhart, Michael Schneider, Matthias W. Beckmann, Christian Bogdan, Roman G. Gerlach, Lina Gölz

**Affiliations:** a Department of Orthodontics and Orofacial Orthopedics, Universitätsklinikum Erlangen Friedrich-Alexander-Universität (FAU) Erlangen-Nürnberg, Erlangen, Germany; b Department of Oral and Cranio-Maxillofacial Surgery, Friedrich-Alexander-Universität (FAU) Erlangen-Nürnberg, Erlangen, Germany; c Department of Pediatric and Adolescent Medicine, Universitätsklinikum Erlangen Friedrich-Alexander-Universität (FAU) Erlangen-Nürnberg, Erlangen, Germany; d Department of Pediatrics, Universitätsklinikum Erlangen Friedrich-Alexander-Universität (FAU) Erlangen-Nürnberg, Erlangen, Germany; e Department of Gynecology and Obstetrics, Comprehensive Cancer Center (CCC) Erlangen-EMN, Universitätsklinikum Erlangen Friedrich-Alexander-Universität (FAU) Erlangen-Nürnberg, Erlangen, Germany; f Mikrobiologisches Institut – Klinische Mikrobiologie, Immunologie und Hygiene, Universitätsklinikum Erlangen, Friedrich-Alexander-Universität (FAU) Erlangen-Nürnberg, Erlangen, Germany; g Medical Immunology Campus Erlangen, FAU Erlangen-Nürnberg, Erlangen, Germany

**Keywords:** Orofacial clefts, neonates, oral microbiome, microbiome maturation, early life dysbiosis

## Abstract

Orofacial clefts (OFC) present different phenotypes with a postnatal challenge for oral microbiota development. In order to investigate the impact of OFC on oral microbiota, smear samples from 15 neonates with OFC and 17 neonates without OFC were collected from two oral niches (tongue, cheek) at two time points, i.e. after birth (T0: Ø3d OFC group; Ø2d control group) and 4–5 weeks later (T1: Ø32d OFC group; Ø31d control group). Subsequently, the samples were analyzed using next-generation sequencing. We detected a significant increase of alpha diversity and *anaerobic* and *Gram-negative species* from T0 to T1 in both groups. Further, we found that at T1 OFC neonates presented a significantly lower alpha diversity (lowest values for high cleft severity) and significantly higher levels of *Enterobacteriaceae (Citrobacter, Enterobacter, Escherichia-Shigella, Klebsiella), Enterococcus, Bifidobacterium, Corynebacterium, Lactocaseibacillus, Staphylococcus, Acinetobacter* and *Lawsonella* compared to controls. Notably, neonates with unilateral and bilateral cleft lip and palate (UCLP/BCLP) presented similarities in beta diversity and a mixture with skin microbiota. However, significant differences were seen in neonates with cleft palate only compared to UCLP/BCLP with higher levels of *anaerobic species*. Our findings revealed an influence of OFC as well as cleft phenotype and severity on postnatal oral microbiota maturation.

## Introduction

Cleft lip and palate (CLP) are the second most common human malformation and show a varying prevalence of approximately 1:700 for cleft lip palate and 1:1,200 for cleft palate only depending on ethnic group, cleft type/side and gender [1]. They present different phenotypes, e.g. bilateral cleft lip and cleft palate (BCLP), unilateral cleft lip and cleft palate (UCLP), Cleft palate only (CPo) and Cleft lip only (CLo) [2]. Orofacial clefts are the result of impaired maxillofacial development processes between the 7th and 12th embryonic week [3]. Due to the variety of cleft types and severities, several classification schemes have been evolved [2,4], e.g. the LAHSHAL classification scheme by Kriens *et al* [5], which is the second-most common and recommended classification scheme due to its adequateness and extensiveness [4]. Depending on cleft severity, neonates with OFC require surgical lip and/or surgical palate closure [6,7] as well as presurgical infant orthopedics (PSIO) with palate plates [8], which aim to control growth, ensure feeding and normalize function [7,9]. Despite PSIO treatment, feeding difficulties [10,11] can still occur in neonates with OFC requiring nutrition interventions like Haberman feeder [12,13].

Physiologically, the oral and nasal cavity are solely connected over the nasopharyngeal zone and separated by the hard and soft palate as well as the alveolus and the upper lip; however, depending on the severity of the cleft, both cavities can be joined over smaller to larger areas [2]. This could lead to an unphysiological communication of the oral and nasal microbiome. The oral microbiota displays the second-largest human microbiota after the gut and is composed of up to 775 bacterial taxa in adults including 57% named species, 30% uncultivated species and 13% cultivated unnamed taxa with a provisional name based on the 16S rRNA sequence [14]. Considering initial maturation, some authors showed that the intrauterine environment is sterile indicating that the maturation of the human microbiota begins after birth [15,16], while others demonstrated similarities between the neonatal oral and mothers placental microbiota [17,18] indicating that development of oral microbiota begins during pregnancy. The oral cavity contains anatomically different niches, which partly develop in the embryonic phase and fetal period, e.g. the tongue (T), the soft and hard palate (HP), the cheeks (C) and sublingual area (U), and partly after birth and during childhood, e.g. teeth, the gingival sulcus with the gingival crevicular fluid (GCF) and dental plaque (P). Interestingly, in young periodontal healthy adults, a clustering into three microbial metaniches was detected that can be explained by anatomical proximity, function, salivary glands and saliva flow rate [19]. Regarding neonates with orofacial clefts, it has been shown that 17-month-old infants with non-syndromic OFC presented a lower oral microbial diversity and an altered microbial composition compared to controls using next-generation sequencing after surgical palate closure, however without data prior to surgery [20]. Regarding different cleft types, distinct oral bacterial species were investigated using culture analyses in neonates with CLP and CPo at two time points (1–2 and 8–18 weeks after birth) and significant differences were seen between the cleft types and between the time points, yet a control group was lacking [21]. Contemplating possible changes before and after surgical cleft closure, a decrease of certain bacterial genera was seen in CLP neonates after palate closure using culture analyses and the detection of *beta-hemolytic streptococci* correlated with increased risk for palatal dehiscence; however, a control group was missing in this study [22]. Moreover, in 10-year-old adolescents with CLP, it has been shown that the abundance of specific bacterial species prior to alveolar bone grafting correlated with post-surgical inflammation at operative sites [23]. Further, there is some evidence that dysbiosis of the gut microbiome in neonates may be associated with vulnerability for inflammatory diseases in childhood [24]. Hence, the disturbances of oral microbiome development may be a risk factor for early-life dysbiosis, inflammation and in consequence might be linked to adverse surgery outcomes and dysbiotic inflammatory diseases later in life. Adverse surgical outcome is a major problem in cleft palate surgical with a 22% prevalence of palatal fistulas requiring secondary palate closure in about 49% of these cases [25] as well in secondary alveolar bone grafting were 2–7.4% of grated sites were fully adsorbed with a success rate of solely 60–70% [23,26,27].

Yet, studies investigating an early impact of oral microbiota maturation in neonates with OFC compared to an age-matched control group and regarding oral niches using next-generation sequencing (NGS) are missing. The knowledge about possible early developmental alterations in oral microbiota in neonates with different CLP types might be crucial for future studies investigating risk factors for wound healing disorders within the first year of life and oral inflammatory diseases even later on. Therefore, the aim of our study was to analyse the impact of OFC, cleft phenotype and severity on oral microbiota maturation compared to controls considering two time points, 2–3 days and 4–5 weeks after birth, using NGS. We hypothesize that OFC malformation is a risk factor for oral dysbiosis.

## Methods

This study was designed as a prospective observational study and has been approved by the local ethics committee of the Friedrich-Alexander-University Erlangen-Nürnberg (Krankenhausstraße 12, 91054 Erlangen, Vote number: 168_20 B, 28.04.2020) prior to the beginning of the study. The trial was performed in accordance with the declaration of Helsinki and the STROBE guideline checklist [28]. A total of 107 patients were recruited following predefined inclusion criteria: 1) Neonates with non-syndromic orofacial cleft OR neonates without orofacial cleft and 2) written informed consent by the parents and/or legal guardians. Exclusion criteria were defined as: 1) Neonates with syndromic cleft lip and palate; 2) Preterm birth (<37 weeks gestational age); 3) neonates with underweight at birth (<2,500 g); 4) neonates with systemic and metabolic diseases that were shown to impact oral health, e.g. diabetes, metabolic syndrome, liver disease, cardiovascular disease, adverse pregnancy outcomes and cancer [29–31]; and 5) revoked written informed consent by the parents and/or legal guardians. Two informed consent forms for participation in the trial and for utilization of soft tissue smear samples, data protection sheets and information material explaining the study in adequate language were provided. Written informed consent forms and data protection sheets by the parents and/or legal guardians were mandatory for enrolment in the trial. To collect information about patient-specific information, written questionnaires were given to the parents and/or legal guardian to collect information about neonates’ clinical parameters including weight and height at birth, nutrition protocol, intake of antibiotics and/or supplements as well as to collect information about the mother including type of birth (vaginal, caesarian), premature rupture of membranes (PROM) and intake of antibiotics (Table 1). These questionnaires were handed out at the first consultation after birth (T0) and collected 4–5 weeks later (T1). At T1, the parents and/or legal guarding were asked whether changes in the intake of antibiotics and/or supplements or the nutrition protocol occurred.

**Table 1.** Study population.

#	Age at T0 (d)	Age at T1 (d)	Gender	Weight T0 (g)	Height T0 (cm)	Type of birth	PROM	Antibiotics	Nutrition^1^
Characteristics of the control group									
LKGc-001	2	36	f	3350	52	c	0	ABM	0
LKGc-002	2	34	m	3800	53	c	0	ABM	0
LKGc-003	2	27	m	4120	55	c	0	ABM	0
LKGc-004	2	23	f	3150	50	c	1	ABM	0
LKGc-005	2	32	m	3340	50	v	0	noABM	0
LKGc-008	3	29	f	3180	51	v	1	noABM	0
LKGc-009	2	35	m	4030	54	v	0	noABM	0
LKGc-010	2	38	m	3640	53	v	0	noABM	0
LKGc-011	2	31	m	3050	50	v	1	noABM	0
LKGc-012	2	20	f	2930	50	v	1	noABM	0
LKGc-013	3	32	m	3670	54	v	1	noABM	0
LKGc-015	3	34	f	3200	50	v	1	noABM	0
LKGc-016	2	42	m	3940	53	c	1	ABM	0
LKGc-018	2	24	f	3570	54	v	0	noABM	0
LKGc-020	3	36	f	4200	56	c	0	ABM	0
LKGc-021	3	22	m	3480	54	c	0	ABM	0
LKGc-022	3	24	m	2950	50	v	0	noABM	0
Characteristics of the study group (CLP)									
LKG-001	7	24	m	2590	48	v	0	noABM	2
LKG-002	n.d.	19	m	3130	44	c	0	ABM	2,*4*
LKG-003	3	38	m	3040	51	c	0	ABM	2
LKG-004	5	n.d.	f	2980	51	v	0	noABM	0
LKG-005	2	37	f	2940	51	v	0	noABM	1
LKG-007	2	29	m	3220	51	v	0	noABM	1
LKG-009	3	29	m	3240	49	v	0	ABM	3
LKG-010	3	38	m	3320	51	c	0	ABM	3,*4*
LKG-011	3	34	m	3350	53	v	0	noABM	1
LKG-012	3	25	m	3900	51	v	0	noABM	2,*4*
LKG-014	2	31	m	3120	47	c	0	ABM	2
LKG-015	1	22	f	2800	51	v	0	noABM	2
LKG-016	11	39	f	3120	51	v	0	noABM	1,*4*
LKG-017	14	40	m	2860	50	v	0	noABM	3
LKG-018	8	34	f	3890	51	v	0	noABM	0
Special characteristics of the study group: classification, severity, type of treatment									
#	Etiology	BCLP	UCLP*	CPo	CLo	LAHSHAL Code [2]	LAHSHAL Severity [3]	Severity Score [4]	pAM
LKG-001	ps	0	1	0	0	- - - SHAL	0002222	8	1
LKG-002	s	0	0	1	0	- - hSh - -	0012100	4	0
LKG-003	ps	0	1	0	0	- - - SHAL	0002222	8	1
LKG-004	s	0	0	1	0	- - hSh - -	0012100	4	0
LKG-005	s	0	0	1	0	- - HSH - -	0022200	6	1
LKG-007	ps	0	1	0	0	- - - SHAl	0002221	7	1
LKG-009	ps	1	0	0	0	lAHS - - l	1222001	8	1
LKG-010	ps	1	0	0	0	LAHSHAL	2222222	14	1
LKG-011	ps	0	1	0	0	LAHS - - -	2222000	8	1
LKG-012	ps	1	0	0	0	LAHSHAL	2222222	14	1
LKG-014	ps	0	1	0	0	lAHS - - -	1222000	7	1
LKG-015	ps	1	0	0	0	laHSHAL	1122222	12	1
LKG-016	s	0	0	1	0	- - HSH - -	0022200	6	1
LKG-017	s	0	0	1	0	- - HSH - -	0022200	6	1
LKG-018	p	0	0	0	1	la - - - - -	1100000	2	0

0 = no, 1 = yes, s = cleft of the secondary palate, *p* = cleft of the primary palate, ps = cleft of the primary and secondary palate, d = days, g = grams, cm = centimeter, f = female, *p* = male, n.d. = not done, c = Caesarian section, v = vaginal, PROM = premature rupture of membranes, noABM = no antibiotic intake of mother intrapartum, ABM = antibiotic intake mother intrapartum, pAM = passive alveolar molding, T0 = time point T0 after birth, T1 = Time point T1 4–5 weeks after birth, BCLP = bilateral cleft lip palate, UCLP = unilateral cleft lip palate, CPo = Cleft Palate only, CLo = Cleft Lip only.1 0 = breastfeeding, 1 = bottle feeding breast milk, 2 = bottle feeding partly breast milk, partly artificial formula, 3 = bottle feeding artificial formula, 4 = postnatal tube feeding for <1 week (=T0); 2 LAHSHAL Code: minus sign (-) =not affected, small letter = incompletely affected, capital letter = completely affected; 3 LAHSHAL Severity: 0 = not affected, 1 = incompletely affected, 2 = completely affected; 4 Severity Score: sum of the LAHSHAL Severity

### Sample collection

**Figure 1.**
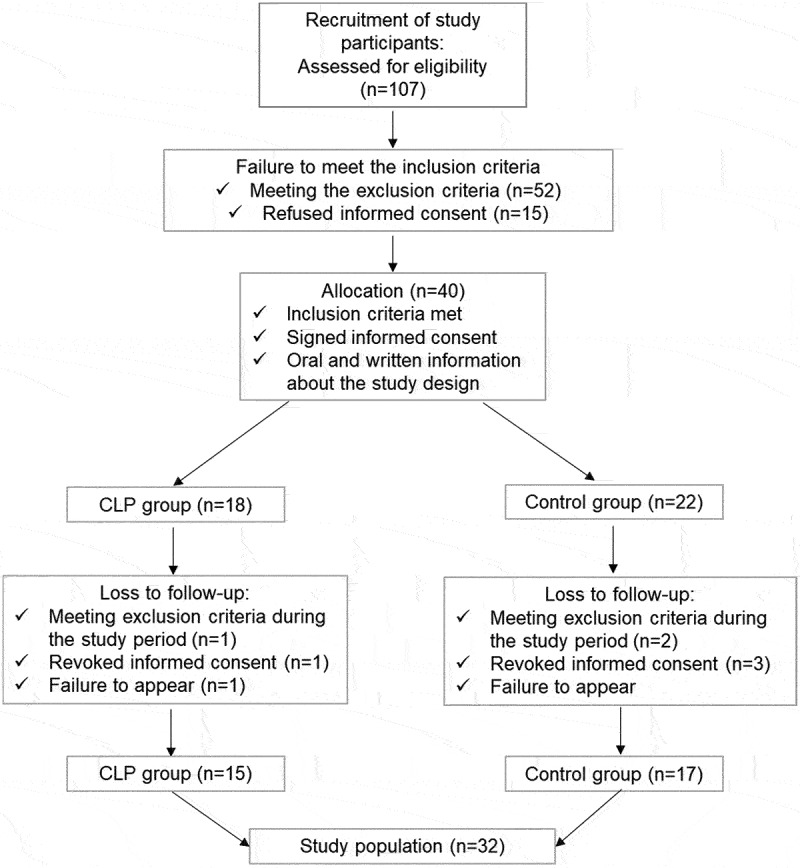
Flow of study participants.

After obtaining informed consent, 158 samples were collected from 40 study participants at two time points (T0, T1) from two different oral niches from each study participant from June 2020 to June 2021 by one experienced orthodontist specialized in treatment of neonates with CLP [9] and trained in preliminary experiments in taking of oral samples [19,32,33]. Due to drop-outs, 32 samples were excluded from further analyses (Figure 1). Therefore, the final sample size accounted to 126 samples from 32 study participants (Figure 1). The oral niches tongue (T) and cheek (C) were chosen as representatives of two of the three previously detected metaniches [19]. The third metaniche (gingival crevicular fluid and plaque) [19] is not expected in neonates since tooth eruption and congenital eruption cysts rarely occur in neonates [34]. Soft tissue samples were collected using sterile swabs patting over the area T (middle and anterior part) and C (right side) for about 10 s and parents were instructed not to feed the neonate up to two to three hours before sample collection. Regarding the cleft lip and palate (CLP) group, the first time point (T0) was defined as the first consultation at the Department of Orthodontics and Orofacial Orthopedics, which is a regular appointment for clinical investigation (T0: median 3d CLP group). On this appointment, orthodontists specialized in the treatment of newborns with orofacial cleft investigate the newborn, make a diagnosis and decide whether presurgical treatment with a palate plate is needed. In case of treatment need with presurgical infant orthopedics (PSIO), e.g. treatment with passive palate plates (passive alveolar molding = pAM) (Table 1), neonates have regular appointments every 4–6 weeks. The second time point (T1) was defined as a regular control appointment for the CLP group 4–5 weeks after birth (T1: median 32d CLP group). Treatment of CLP neonates does not include intake of antibiotics or other medications or surgical interventions within the first weeks of life (T0 – T1). Considering the control group, the first time point (T0) was defined as the second (‘U2’) routine investigation for children after birth (called ‘U’ investigations) performed in the Department of Gynecology and Obstetrics (median Ø2 d control group). The second time point (T1) was defined as the third (‘U3’) routine investigation at local pediatricians for the control group after birth (T1: median 31d control group). All samples were collected in sterile tubes and stored on dry ice within seconds after sample collection. For interim storage, all samples were frozen at −20°C for a maximum of 5 days under maintenance of an uninterrupted cold chain. Afterwards, all samples were frozen and stored at −80°C (CryoCube unit) in the research laboratory of the Department of Orthodontics and Orofacial Orthopedics. Further processing and DNA isolation of samples was performed at the research laboratory of the Department of Orthodontics and Orofacial Orthopedics. Microbiota analyses were conducted in the Institute of Clinical Microbiology, Immunology and Hygiene.

### Classification of cleft severity

The LAHSHAL classification scheme by Kriens *et al* [5]. uses letters to describe the anatomical parts affected by cleft in the following sequence: right lip, right alveolus, right hard palate, soft palate, left hard palate, left alveolus and left lip. Capital letters represent complete clefting of the affected anatomical part (L= lip, A = alveolus, H = hard palate, S = soft palate), while small letters (l, a, h, s) depict incomplete clefting of the affected structure [5]. Anatomical parts that are not affected by clefting are described with a minus sign, minimal clefting with an asterisk and skin band with a plus next to the letter [5], e.g. the LASHSAL code describes complete bilateral cleft lip palate, LAHS – describes complete unilateral cleft lip palate on the right side and – hSh – describes cleft palate only with incomplete cleft of the hard palate and complete cleft of the soft palate. The classification according to LAHSHAL classification was performed by two orthodontists specialized in treatment of neonates with CLP [9] using LAHSHAL classification in daily practice for standard diagnosis. In order to calculate the severity of the clefting (LAHSHAL Severity), we developed a ‘LAHSHAL Severity Score’, which was the sum of the numbers of the LAHSHAL Severity: 0 = not affected, 1 = incompletely affected, 2 = completely affected (Table 1). Hence, the most severe form of clefting, the complete bilaterial cleft lip and palate (BCLP LAHSHAL Code: LAHSHAL), is described with 2,222,222 summing up to LAHSHAL Severity Score 14 (Table 1).

### Outcomes

The primary outcome was to analyse the oral microbiome in neonates with orofacial clefts (CLP group) compared to controls (CTRL group). The secondary outcome was to investigate whether cleft phenotype or severity impact the oral microbiome within the CLP group. Since solely one study participant with CLo was recruited (a very rare CLP formation), this study participant was not included in all comparisons within the CLP group. Further, we wanted to examine whether potential cofounders (birth mode, antibiotics, nutrition) alter the microbiome composition. For investigation of these outcome variables, the oral microbiome was analysed as follows: DNA isolation, amplification of the V1-V3 region of the 16S rDNA and sequencing was carried out as described before [19]. Raw sequencing data are available through the National Center for Biotechnology Information (NCBI) Sequence Read Archive (SRA) under BioProject PRJNA881235 (https://www.ncbi.nlm.nih.gov/sra/PRJNA881235). Raw reads were filtered and processed as described before [19]. For merging read pairs and OTU clustering with the USEARCH v11 algorithm [35], sequences were re-multiplexed using a Perl script (I. Lagkouvardos, available from www.imngs.org) and submitted to the IMNGS server [36]. After removal of five samples due to low (below 2,000) merged read counts, the read median of the remaining samples was 36,908 (range: 2,402–1,629,504). Taxonomic classification of the identified OTUs was performed using the SILVA alignment, classification and tree service [37] with SILVA database release 138.1 and NCBI nucleotide BLAST [38]. The resulting OTU table and OTU sequences are available as supplementary material (Supplementary Table s1, 2). Further analyses were carried out within R v4.2.0 [39] by importing the modified OTU table, the SILVA phylogenetic tree and metadata using the package phyloseq [40]. Phyloseq functions were used to filter the data, calculate and visualize diversity and to perform ordination. For ordination, we used log-transformed counts to stabilize variance and calculated distances based on the generalized UniFrac algorithm (GUniFrac package for R). Differential abundance analysis was carried out using EdgeR [40], limma [41] and voom [42] with ggplot2 [43] for visualization. BugBase [44] was used to estimate the proportion of ‘high level phenotypes’ such as *Gram-positive*, *Gram-negative*, *anaerobes*, *aerobes*, *biofilm-formers*, *stress tolerant strains* or *bacteria with mobile elements*. For this analysis, the OTU table and -sequences were imported in QIIME 2 v2021.11 [45] and taxonomic classification was performed with VSEARCH [46] against the GreenGenes v13.8 16S rRNA database [47] with 97% similarity threshold. The data were exported as biom (v1) file and finally submitted to the BugBase server (https://bugbase.cs.umn.edu/).

### Statistics

The statistical analyses of the oral microbiome was performed with R v4.2.0 using packages vegan [39] and pairwiseAdonis (https://github.com/pmartinezarbizu/pairwiseAdonis). Statistical analysis of beta diversity was carried out with permutational multivariate analysis of variance (PERMANOVA) as implemented in the adonis2 and anova.cca functions of the vegan package for R. Similar dispersion of the analyzed groups was tested with betadisper and permutest functions (vegan) and subsequent Tukey’s HSD test. For investigation of different high-level phenotypes, the identified microbial communities were subjected to BugBase [44] analysis, which is based on PICRUSt [48]. The statistical investigation of the study population was performed with SPSS statistical software (IBM SPSS Statistics Version 28.0.0.0 (190)). The data sets were analyzed using the Mann-Whitney U-test and chi-square test. Differences were considered significant with *p*-values <0.05.

## Results

### Characteristics of the study population

A total of 107 patients were examined for eligibility. In total, 67 individuals were not included in the study due to not meeting the inclusion criteria (*n* = 52) or refused informed consent (*n* = 15). After eligibility screening, a total of 40 study participants were enrolled in this study and divided into two groups (Figure 1). The study group, neonates (*n* = 18) with orofacial clefts, was recruited at the Department of Orthodontics and Orofacial Orthopedics. The control group, neonates without orofacial clefts (*n* = 22), was recruited at the Department of Gynaecology and Obstetrics. Dropouts were registered due to the following reasons: 1) Meeting the exclusion criteria during the course of the study, e.g. diagnosis of a syndrome or acute systemic or metabolic disease (CLP group: *n* = 1, control group: *n* = 2), 2) revoke of consent by the parents and/or legal guardians (CLP group: *n* = 1, control group: *n* = 3), or 3) Failure to appear to the consultation and study appointments (CLP group: *n* = 1, control group: *n* = 0) (Figure 1). In total, the dropout rate was 15% (*n* = 8 in total) with a final sample size of 15 study participants in the study group and 17 study participants in the control group (Figure 1). The final study group comprised of five study participants with BCLP, four with UCLP, 5 with CPo and one study participant with CLo (*n* = 1) (Table 1).

Characteristics of the CLP and control group were collected and compared statistically (Table 2). No differences were seen with regard to gender, type of birth or intake of antibiotics (Table 2). However, statistical differences were seen regarding birth weight and height (Table 2). Since the first consultation in the CLP group earliest takes place when transport of neonates is possible, the median age for the CLP was 1 day elder than in the control group at T0 (*p* = 0.046). However, differences at T1 were not detectable (Table 2). Further, neonates with orofacial cleft showed specific characteristics with regard to cleft type, cleft severity and need for treatment (Table 1). While neonates with severe cleft severity are in need of treatment with palate plates (pAM) [49], neonates with minor cleft severity sometimes present no feeding problems and are therefore not in need of treatment and require less appointments (Table 1, LKG-002/-004/-018). Hence, two neonates with incomplete CPo (LKG_002 and LKG_004) required only one appointment and only one time point was collected (LKG_002 only T1: late first consultation with no treatment need; LKG_004 only T0: no need for further treatment).

**Table 2.** Statistical comparison of study and control group characteristics.

Characteristics		CLP group (*n* = 15)	Ctrl group (*n* = 17)	p-value
Age	T0 (d) *	3 (2–7.25)	2 (2–3)	0.025^a^
	T1 (d) *	32 (24.75–38)	31 (24–35.5)	0.633^a^
Gender	f:m ratio (n)	5:10	7:10	0.826^a^
Weight	T0 (g) *	3120 (2920–3328)	3480 (3165–3870)	**0.019**^a^
Height	T0 (g) *	51 (49.75–51)	53 (50–54)	**0.029**^a^
Type of birth	v:c ratio (n)	11:4	10:7	0.396^a^
Antibiotics	noAB:ABM:ABN	9:5:1 (60)	10:7:0 (59)	0.531^b^
Nutrition	BM:BMB:MF:AF:TF	2:3:4:2:4	17:0:0:0:0	**<0.001**^b^

d = days, g = grams, cm = centimeter, f = female, *m* = male, *m* = number; v = vaginal, c = caesarian, noAB= no antibiotic intake of neonates or mother before birth, ABM = antibiotic intake mother intrapartum, ABN = antibiotic intake neonate after birth, T0 = time point T0 after birth, T1 = Time point T1 4–5 weeks after birth; BM = breastfeeding; BMB = breast-milk bottle feeding; MF = mixed baby food; AF = artificial baby food; TF = tube feeding; * median (IQR); a Mann-Whitney Test; b chi-square Test.

### Oral microbial composition in neonates with OFC and controls after birth and within the first weeks of life

**Figure 2.**
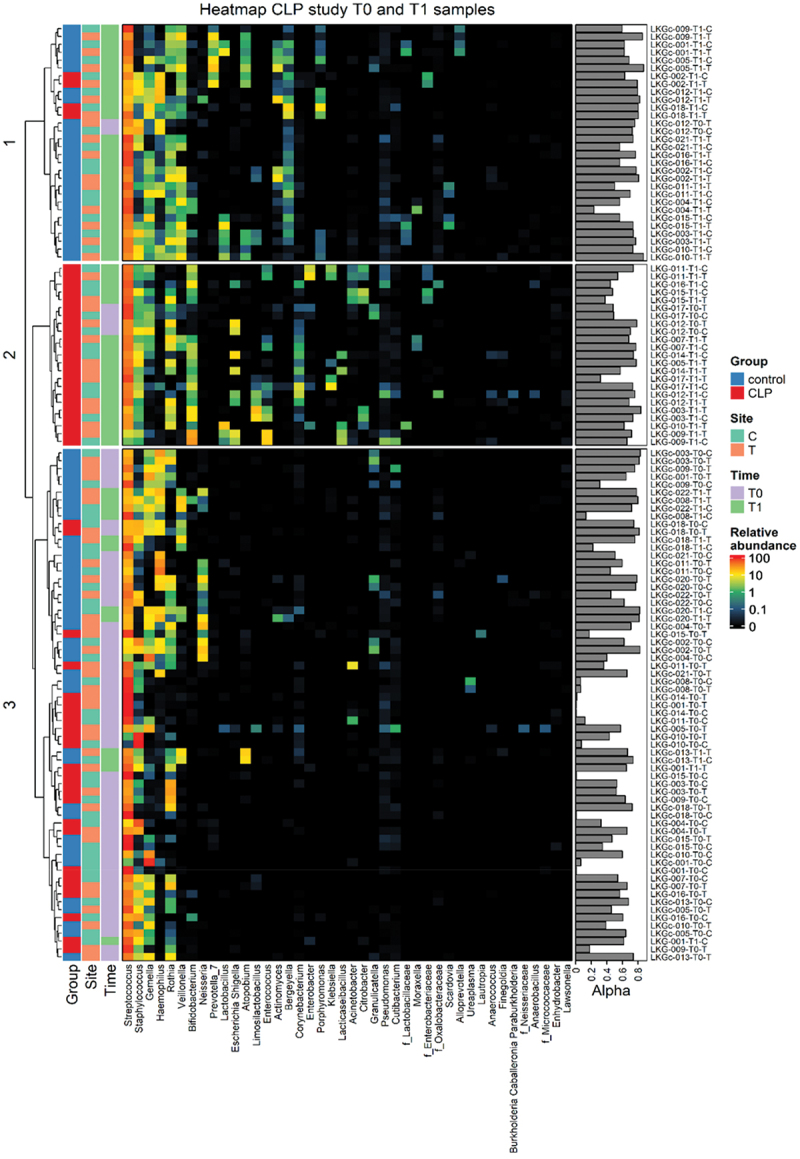
Dendrogram and genus frequencies plot. A dendrogram based on generalized UniFrac distances is given for each individual sample (excluded samples see Material & Methods) collected from two groups (red: cleft lip palate (‘LKG’/CLP) group; blue: control (‘LKGc’) group), two niches (orange: tongue T; turquoise: cheek C) and two time points (purple: T0, green: T1). A heatmap representing relative abundances (black = no abundance; red = highest abundance) of microbial genera found in individual samples. Samples are ordered vertically according to the dendrogram, while microbial genera are presented horizontally with left to right decreasing mean relative abundance in all samples. The Simpson diversity index representing alpha diversity (Alpha) is shown on the right side for each sample with sample identifier next to it.

In total, 123 oral samples from two groups (15 neonates with orofacial clefts/cleft lip palate ‘CLP group’ vs. 17 neonates without orofacial clefts/control ‘CTRL group’), two time points, i.e. at first consultation after birth (T0: Ø3d CLP; Ø2d control) and 4–5 weeks later (T1: Ø32d OFC; Ø31d control) and two different oral niches (T, C) were collected and used for microbiota analyses. The following samples were excluded from further analyses due to low merged read counts: LKG-005-T0-C, LKG-005-T1-C, LKG-016-T1-T, LKGc-016-T0-C and LKGc-016-T0-T. After removal of these samples, the read median was 36,908. Microbiota analyses of the remaining 118 samples revealed four microbial phyla, nine classes, 20 orders, 30 families, and 39 genera derived from 117 operational taxonomic units (OTU). We used generalized UniFrac distances of log-transformed genus counts to calculate a dendrogram reflecting the differences in beta diversity of the samples (Figure 2). There was neither clustering observed with respect to the sampling sites tongue and cheek nor with respect to a combination of groups (CLP vs. control) and niches. However, the dendrogram showed clustering into three branches with the lower branch mainly formed by samples of time point T0 of both groups (Figure 2). Within this branch, a trend to a separation into two clusters formed by the CLP and control group can be seen, however, those clusters were not clearly distinguishable (Figure 2). The middle branch represents a distinct cluster for the CLP group at time point T1 without separation into oral niches (Figure 2). Control samples from time point T1 including both niches clustered in the upper branch (Figure 2). Here, also two patients of the CLP group clustered: LKG-002 and LKG-018, both presenting only mild phenotypic manifestations (Table 1). Since LKG-018 was the only neonate affected by CLo and, therefore, the only patient without clefting of intraoral structures (soft/hard palate and alveolus) and a potential mixture of nasal and oral microbiota, it was excluded in further investigations referring to the complete CLP group. LKG-002 was still included in further investigations due to the affection of intraoral structures (soft/hard palate).

After birth (T0), oral microbial composition in neonates from both groups was dominated by the following genera (CLP; control): *Streptococcus* (68.03%; 59.18%), *Staphylococcus* (15.84%; 8.70%), *Gemella* (4.88%; 12.17%) and *Rothia* (8.36%; 3.67%). Notably, in one CLP neonate (LKG_014_T0), a mono colonization of *Streptococcus* was found (Figure 2).

Within the first weeks of life (T1), high abundance of *Streptococcus* (65.01%; 66.58%), *Staphylococcus* (4.72%; 4.34%), *Gemella* (1.97%; 4.84%) and *Rothia* (4.36%; 2.68%), the subsequent genera were still detected in both groups (CLP; control) (Figure 2). However, a higher variety of genera was found in both groups. The control group presented elevated levels of *Haemophilus* (6.21%), *Veillonnella* (7.18%), *Lactobacillus* (1.06%), Prevotella 7 (1.93%), *Atopobium* (1.61%) and *Actinomyces* (1.54%) (Figure 2). The CLP group showed increased levels of *Bifidobacterium* (7.94%), *Neisseria* (3.88%), *Escherichia-Shigella* (1.99%), *Lactobacillus* (1.60%), *Limosilactobacillus* (2.34%), *Enterococcus* (2.45%), *Bergeyella* (1.20%) and *Corynebacterium* (1.15%) (Figure 2). Differential analyses comparing beta diversity on genus levels between the CLP and control group is given in Figure 7.

Taken together, the oral microbial composition of CLP and control neonates after birth (T0) was similar and dominated by *Streptococcus* spp. without distinct clustering according to group or site. Concerning both groups after 4–5 weeks of life (T1), a greater microbial variety and distinct formations of oral microbiota were seen for neonates with orofacial clefts and neonates without CLP based on abundance of microbial genera. Notably, metaniche-characteristics [19] were not seen in both groups after birth and within the first weeks of life.

### Alpha diversity in neonates with OFC and controls after birth and within the first weeks of life considering different cleft types and exogeneous factors

Alpha diversity is measured as species richness (count of number of operational taxonomic units (OTUs)) or evenness (relative abundance of OTUs) present in one site of an individual sample [50,51].

**Figure 3.**
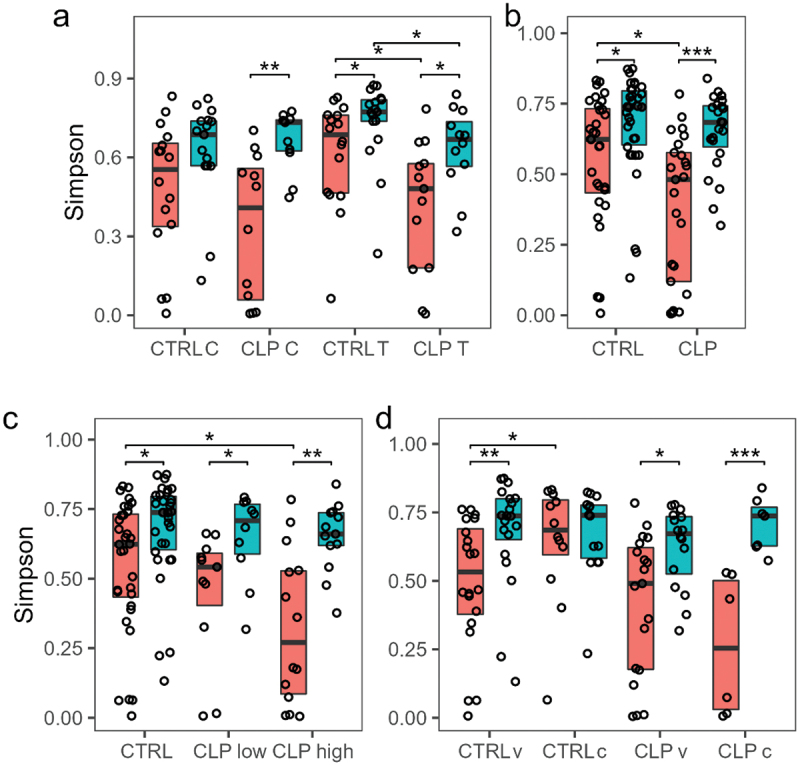
Alpha Diversity. Box and scatter plots of Simpson diversity is given for both time points T0 and T1 (orange = T0; turquoise = T1) for (a) cleft lip palate (CLP) vs. control (CTRL), tongue (T) vs. cheek (C); (b) CLP vs. CTRL; (c) CTRL vs. CLP with low Severity score 2–7 (CLPlow) vs. CLP with high Severity score 8–14 (CLPhigh); (d) CLP vs. control CTRL considering different type of birth vaginal (v) vs. caesarian birth (c). T1). Simpson diversity of individual samples was calculated based on OTU counts (dots). Boxes show 25^th^ percentile, median and 75^th^ percentile. Wilcox tests were used to calculate pairwise comparison statistics as indicated. Differences were considered statistically significant for *p* < 0.05 (*), *p* < 0.01 (**), *p* < 0.001 (***).

**Figure 4.**
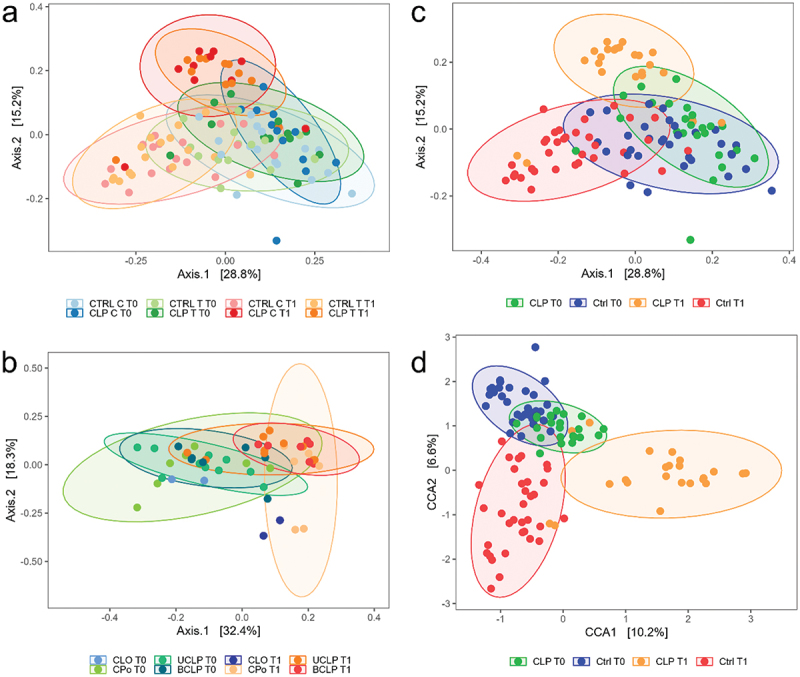
Beta Diversity. Multidimensional scaling (MDS) plots based on generalized UniFrac distances of log-transformed genus counts are shown for (a) cleft lip palate (CLP) vs. control (Ctrl), tongue (T) vs. cheek (C) and time points T0 vs. T1; (b) Cleft Lip only (CLo) vs. Cleft Palate only (CPo), unilateral cleft lip palate (UCLP), bilateral cleft lip palate (BCLP) and T0 vs. T1 and (c) CLP vs. Ctrl and T0 vs. T1. In (d) a Canonical Correspondence Analysis (CCA) of log-transformed genus counts constrained to group (CLP, Ctrl) and time point (T0, T1) is depicted.

Together with patient LKG-002, we found patient LKG-018 to cluster with the control group at T1 (Figure 2). Because of its relatively mild CLP manifestation (LAHSHAL: la - - - - -) and no affection of intraoral structures we excluded patient LKG-018 in the following ‘CLP group’ versus ‘control group’ comparisons to facilitate the identification of CLP-specific differences. The CLo patient was solely included for the presented analyses for comparison between cleft types (Figure 4 +6). Considering both sampling niches for calculating the Simpson diversity index as a measure for alpha diversity, we observed a significantly lower alpha diversity of tongue samples in CLP neonates compared to control neonates at both time points (Figure 3 a). Except for cheek samples of the control group, we further observed a significant increase in alpha diversity from T0 to T1 in all groups (Figure 3 b). To evaluate the impact of CLP severity on a more granular, individual base, we transformed the LAHSHAL code in a severity score using numbers (see Methods section). Interestingly, significant differences were seen at T0 with a higher alpha diversity in control neonates compared to CLP neonates with high severity scores (Figure 3 c). CLP neonates with low severity scores also presented a higher alpha diversity compared to CLP neonates with high severity scores at T0 (Figure 3 c). Regarding birth mode, the control group presented a significantly higher alpha diversity in neonates born via C-section at T0, while no differences were seen for CLP neonates and at T1 for both groups (Figure 3 d) indicating that differences between the control and CLP group are not due the different birth modes.

Summarizing, we demonstrated a significant increase of alpha diversity from T0 to T1 in both groups. However, the presence of orofacial clefts (CLP group) resulted in lower increase from T0 to T1 and overall reduced alpha diversity in the niche tongue at both time points and in both niches combined at T0 compared to controls. This effect was most pronounced in patients with high cleft severity which seems to have a detrimental effect on alpha diversity.

### Ordinate analyses using multidimensional scaling and constrained correspondence analyses

Beta diversity is evaluated as the variance in species composition (the identity of OTUs observed) among samples within a habitat (group, site) [50,51].

In order to investigate beta diversity, ordination using multidimensional scaling (MDS) based on generalized UniFrac distances of log-transformed genus counts were performed visualizing 44% (Figure 4 a and c) and 50.7% (Figure 4 b) of whole data variability. Applying permutational multivariate analysis of variance, no significant differences were seen with a combination of sample niche (T vs. C), patient group and time point (Figure 4 a) variables. However, using the same test significant differences (*p* = 0.001) were obtained with the combination of group and time point variable only. From this we concluded that, in contrast to the adult oral microbiome with defined metaniches [19], there is a more uniform distribution of microbial species in the oral cavity at this age. Consequently, group differences were analyzed with sampling niches combined which resulted in two samples per patient and time point when referring to beta diversity. We observed only minor differences in beta diversity for both groups at T0 (pairwise adonis2 p adj. = 0.018) with the two clusters largely overlaying each other (Figure 4 c). However, we could show more significant differences between the CLP and the control group at T1 (pairwise adonis2 p adj. = 0.006) with noticeable separation of both clusters (Figure 4 c). To demonstrate this more clearly, we performed constrained correspondence analysis (CCA) of the same data with constriction to group and time point (Figure 4 d). Representing 16.8% of whole data variability, a clear separation of the CLP group from the control was evident at T1 (Figure 4 d). In contrast, the clusters of CLP and control group at T0 were in close proximity to each other and exhibited intersecting areas with the cluster of control group samples at T1 (Figure 4 d). A permutation test proofed significant (*p* = 0.001) for both depicted constrained axes. Notably, the clusters of the UCLP and BCLP group presented almost absolute overlapping at T0, while the CPo group was slightly differentiated from all other categorical groups (Figure 4 b). At T1, the CPo group showed cluster separation with only some overlap with the UCLP and BCLP group (Figure 4 b). Interestingly, the UCLP and BCLP group showed crisscrossing areas at T0 and formed a distinct group with almost completely superimposed confidence ellipses at T1 (Figure 4 b). Solely for comparison between cleft types, the CLo patient (LKG-018) was included presenting similar beta diversity compared to all other groups at T0 and a slight, but not significant separation from all other groups at T1 (Figure 4 b).

To summarize, we detected significant differences in beta diversity between T0 and T1 in both groups. Interestingly, cluster segregation was seen for the CLP group at T1. Moreover, beta diversity detected in UCLP and BCLP was similar at T1, however, the CPo group presented distinct differences at T1. Similar to alpha diversity, no significant differences depending on sampling niche were observed with regard to beta diversity.

### High level phenotype analyses

**Figure 5.**
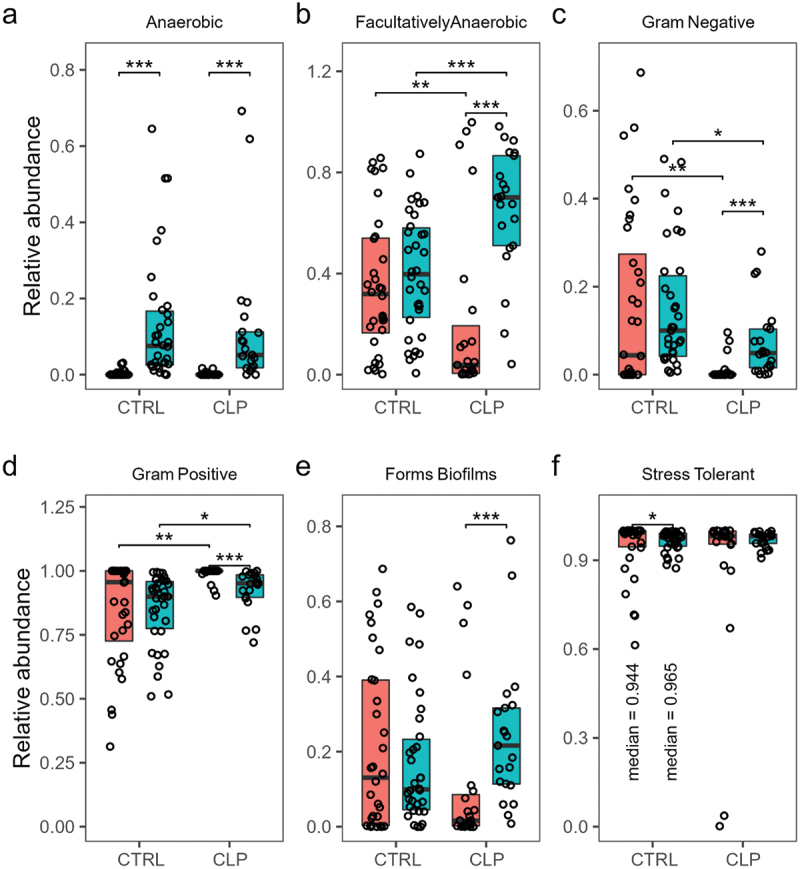
High level phenotype analysis. Relative abundance is given for cleft lip palate (CLP) vs. control (CTRL) and time points T0 (orange colour) vs. T1 (turquoise colour) regarding (a) aerobic, (b) facultative anaerobic, (c) anaerobic, (c) Gram-negative, (d) Gram-positive, (e) biofilm forming and (f) stress tolerant species. For the significant different groups in (f) median relative abundances are given to aid readability. Wilcox tests were used to calculate pairwise comparison statistics as indicated. Differences were considered statistically significant for *p* < 0.05 (*), *p* < 0.01 (**), *p* < 0.001 (***).

**Figure 6.**
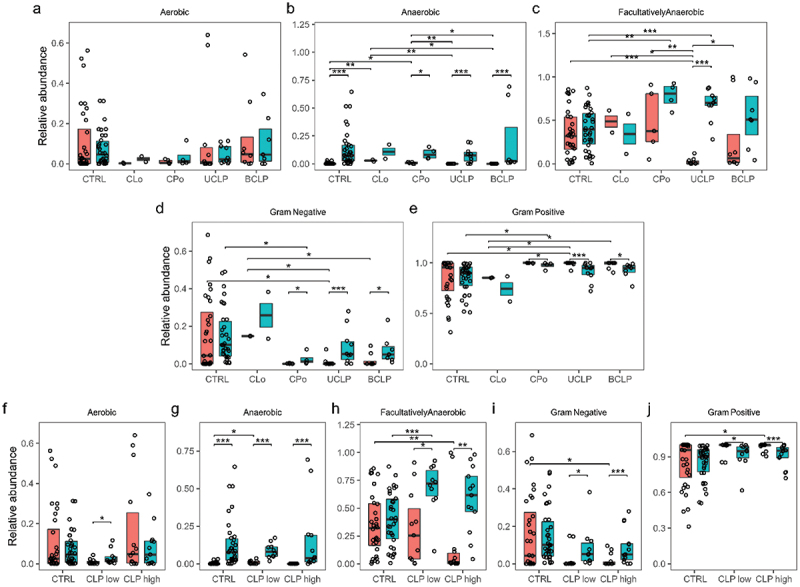
High level phenotype analysis regarding cleft phenotype and severity. Relative abundance is given for different cleft phenotypes Cleft Lip only (CLo), Cleft Palate only (CPo), unilateral cleft lip palate (UCLP), bilateral cleft lip palate (BCLP) and time points T0 (orange colour) vs. T1 (turquoise colour) regarding (a) aerobic, (b) anaerobic, (c) facultatively anaerobic, (d) Gram-negative, (e) Gram-positive species. Relative abundance is given for CLP with low Severity score 2–7 (CLPlow) vs. CLP with high Severity score 8–14 (CLPhigh) and time points T0 (orange colour) vs. T1 (turquoise colour) regarding (f) aerobic, (g) anaerobic, (h) facultatively anaerobic, (i) Gram-negative, (j) Gram-positive species. Wilcox tests were used to calculate pairwise comparison statistics as indicated. Differences were considered statistically significant for *p* < 0.05 (*), *p* < 0.01 (**), *p* < 0.001 (***).

Considering changes from T0 to T1, a significant (*p* < 0.001) increase of *anaerobic species* was seen from T0 to T1 in both groups (Figure 5 a) and regardless of cleft phenotype and severity (Figure 6 b, g). Regarding neonates with orofacial clefts, *Gram-negative species* increased significantly (*p* < 0.001), while in contrast *Gram-positive species* decreased significantly (*p* < 0.001) albeit being still at a high level (Figure 5 d, e). This significant increase of *Gram-negative species* and decrease of *Gram-positive species* was also observed with grouping according to cleft phenotype (CPo: *p* < 0.05; UCLP: *p* < .0.001; BCLP: *p* < 0.05) and severity (low cleft severity *p* < 0.05; high cleft severity *p* < 0.001) (Figure 6 d, e, i, j). The same trend was also found considering the CLo patient (Figure 6 d, e). Controls presented similar but less pronounced (non-significant) proportional changes. Further, a significant (*p* < 0.001) increase of *biofilm forming species* was only seen in CLP neonates from T0 to T1, while *stress-tolerant species* solely increased in control neonates (*p* < 0.05) (Figure 5 e, f).

Comparing neonates with orofacial clefts to controls, *Gram-negative species* were found significantly lower and *Gram-positive species* were significantly higher in the CLP group compared to controls at T0 and T1 (*p* < 0.01, *p* < 0.05) (Figure 5 c, d). Differences between both groups were further seen at T0 with higher levels of *facultative anaerobic* (*p* < 0.001) in controls which was reversed at T1 (Figure 5 b) regardless of cleft phenotype and severity (Figure 6 c).

Comparing different cleft phenotypes and severities, significantly higher levels of *anaerobic species* were seen in controls compared to CLP neonates with low cleft severity (*p* < 0.05) (CLo/CPo: *p* < 0.001/p < 0.05), while CLo/CPo neonates showed higher levels compared to UCLP/BCLP (*p* < 0.01/p < 0.05) neonates at T0 (Figure 6b, g). Moreover, significant differences were seen regarding *facultative anaerobic species* with significantly higher levels in controls compared to UCLP (*p* < 0.001) and compared to CLP neonates with high cleft severity at T0 (*p* < 0.01) (Figure 6c, h). Notably, also CLo/CPo neonates presented higher levels of *facultative anaerobic species* compared to UCLP neonates at T0 (*p* < 0.01/p < 0.05) (Figure 6c). Interestingly, there was a significant (*p* < 0.01/p < 0.05) increase of *facultative anaerobic species* in UCLP and CLP neonates with high and low cleft severity from T0 to T1 resulting in significantly (*p* < 0.001) higher levels of *facultative anaerobic species* in CLP neonates with low cleft severity (CPo: *p* < 0.01) compared to controls at T1 (Figure 6c). Compared to controls, all cleft phenotypes (except for CLo) presented (significantly) lower levels of *Gram-negative* and higher levels of *Gram-positive species* considering both time points (Figure 6 d, e, i, j). Differences were solely significant for UCLP neonates and high severity at T0 (*p* < 0.05) and CPo neonates at T1 (*p* < 0.05) (Figure 6 d, e, i, j). Similar to the control group, the CLo microbiome was enriched for *Gram-negative species* and reduced for *Gram-positive species* at T0 (Figure 6 d, e) with significant differences compared to UCLP and BCLP neonates (Figure 6 d, e).

### Differential abundance analyses at genus level

**Figure 7.**
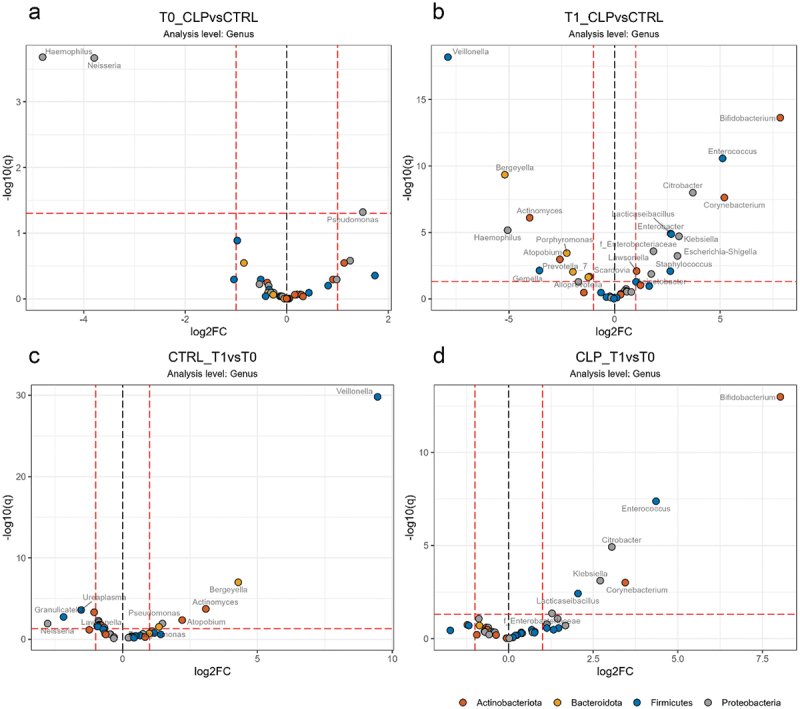
Differential abundance analyses of genera. Volcano plots on genus level is given for a) the cleft lip palate (CLP) vs. control (CTRL) group at T0 and (b) CLP vs. CTRL at T1; d) time point T0 vs. T1 for CTRL group and d) T0 vs. T1 for CLP group. Genus names are given next to colored dots for significant different genera. Dots are colored according to genus phylum as indicated and dashed lines indicate thresholds of significance (>2-fold, adj. p (q) < 0.05).

Consistent with the genus composition seen in the dendrogram (Figure 2), differential abundance analyses at genus level revealed no significant (>2-fold, adj. p (q) < 0.05) differences between both groups at T0 except for higher levels of *Neisseria* and *Haemophilus* in the control (CTRL) group and higher levels of *Pseudomonas* in the cleft lip palate (CLP) group (Figure 7 a). After the first 4–5 weeks of life significant differences were detectable between the groups: the CLP group presented significantly higher abundance of *Enterobacteriaceae* (*Citrobacter*, *Enterobacter*, *Escherichia-Shigella, Klebsiella*), *Enterococcus*, *Bifidobacterium*, *Corynebacterium*, *Lactocaseibacillus*, *Staphylococcus*, *Acinetobacter and Lawsonella* compared to controls (Figure 7 b), while controls showed higher levels of *Veillonella*, *Bergeyella*, *Actinomyces*, *Haemophilus*, *Atopobium*, *Prevotella*, *Porphyromonas*, *Gemella, Alloprevotella* and *Scardovia* (Figure 7 b). Considering changes from T0 to T1, a significant increase of *Veillonella, Bergeyella*, *Actinomyces*, *Atopobium* and *Pseudomonas* was seen in controls (Figure 7 c) and a significant elevation of *Bifidobacterium*, *Enterococcus*, *Citrobacter*, *Klebsiella*, *Corynebacterium* and *Lacticaseibacillus* was seen in the CLP group (Figure 7 d).

### Differential abundance analyses of the CLP group at OTU level

**Figure 8.**
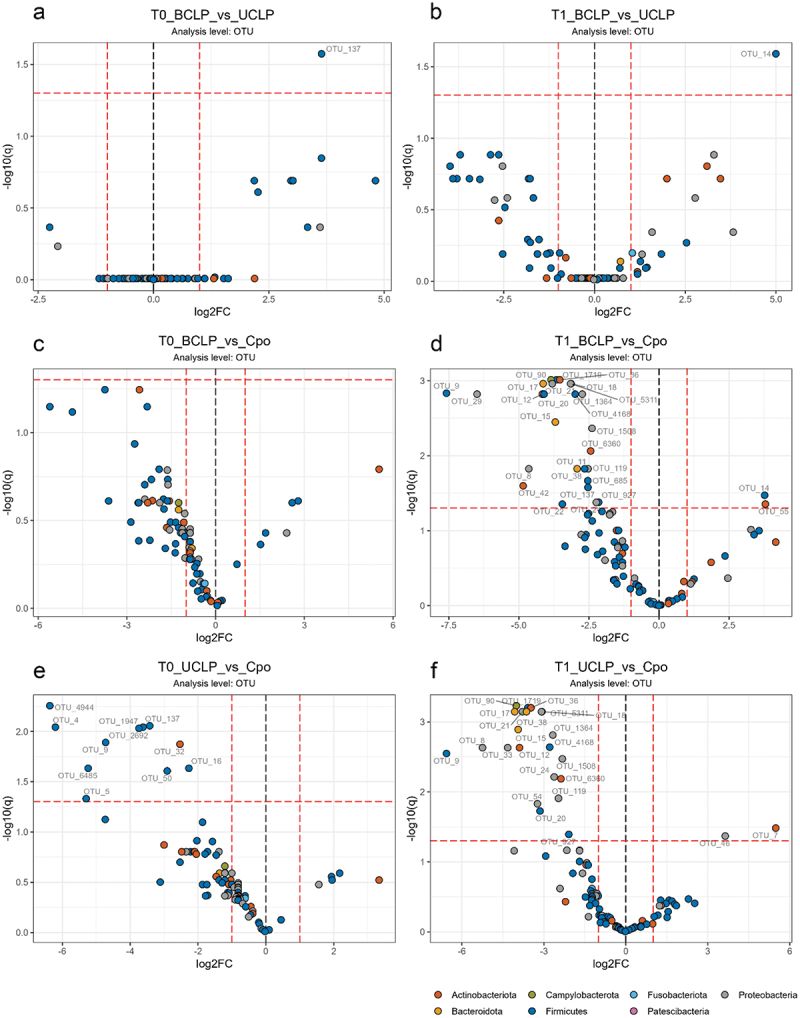
Differential abundance analyses of OTUs. Volcano plots on operational taxonomic (OTU) level is given for a) bilateral cleft lip palate (BCLP) vs. unilateral cleft lip palate (UCLP) at T0; b) BCLP vs. UCLP at T1; c) BCLP vs. CPo at T0; d) BCLP vs. CPo at T1; e) UCLP vs. CPo at T0; f) UCLP vs. CPo group at T1. OTU designations are given next to colored dots for significant different OTUs. Dots are colored according to OTU phylum as indicated and dashed lines indicate thresholds of significance (>2-fold, adj. p (q) < 0.05) between the comparators.

Differential analyses of the CLP group at OTU level presented differences with regard to CLP type (CPo, UCLP, BCLP) considering both time points (T0, T1). At both time points, almost no significant differences were detectable between BCLP and UCLP neonates except for elevated levels of *Streptococcus oralis* (OTU_137) in the BCLP group at T0 (Figure 8 a) and higher proportions of *Lactobacillus gasseri* (OTU_14) at T1 (Figure 8 b). However, differences were more evident between CPo and UCLP/BCLP neonates.

hile at T0 neonates with CPo showed no difference to BCLP, they presented significantly higher levels of *Streptococcus* sp. (OTU_5, OTU_137, OTU_1947, OTU_2692, OTU_4944) compared to UCLP neonates at T0 (Figure 8 c, e). Further, we observed elevated numbers of *Staphylococcus hominis* (OTU_4), *Staphylococcus aureus* (OTU_9), *Granulicatella adiacens* (OTU_50), *Schaalia* sp. (OTU_32) and *Veillonella* sp. (OTU_16) compared to UCLP neonates at T0 (Figure 8 e). At T1, neonates with CPo showed significantly increased abundance of *Klebsiella pneumoniae* (OTU_29), *Prevotella histicola* (OTU_15), *Streptococcus infantis* (OTU_1719), *Streptococcus* sp. (OTU_11, OTU_137, OTU_685), *Haemophilus parainfluenzae* (OTU_8), *Haemophilus haemolyticus* (OTU_24), *Haemophilus* sp. (OTUs: 21, 1364, 1508, 5311), *Staphylococcus aureus* (OTU_9), *Corynebacterium kroppenstedtii* (OTU_42) and *Neisseria sicca* (OTU_119) compared to BCLP (Figure 8 d). Compared to UCLP at T1, we observed for CPo significantly higher numbers of *Campylobacter concisus* (OTU_90), *Staphylococcus aureus* (OTU_9), *Prevotella histicola* (OTU_15), *Prevotella melaninogenica* (OTU_17), *Pseudomonas marginalis* (OTU_33) and *Veillonella atypica* (OTU_20) (Figure 8 f). Compared to CPo at T1, neonates with BCLP exhibited significantly higher abundance of *Lactobacillus gasseri* (OTU_14) and *Bifidobacterium longum* (OTU_55) and neonates with UCLP displayed higher numbers of *Citrobacter freundii* (OTU_46) and *Rothia mucilanginosa* (OTU_7) in the same comparison (Figure 8 d, f).

## Discussion

The aim of the present study was to investigate whether orofacial clefts impact the maturation of the oral microbiome within the first days (T0) and within the first weeks (T1) after birth compared to controls using next-generation sequencing. Further, we wanted to analyze whether cleft severity or phenotype play a crucial role in oral microbiome development. A significant increase of alpha diversity from T0 to T1 was found in both groups. An impact of orofacial clefts on oral microbiome maturation was found at T1 characterized by a significantly lower alpha diversity and significantly higher levels of potential pathogens, e.g. *Enterobacteriaceae*, compared to controls. Further, microbial differences were found between neonates with affection of extraoral structures (UCLP/BCLP) and neonates with cleft palate only (CPo).

Considering oral microbiota after birth, oral genus frequencies showed no significant clustering into CLP and control neonates. Yet, a trend to a distinct microbiome in CLP compared to control neonates can be seen. Accordingly, a relatively homogeneous human microbiome without postnatal clustering was reported [52]. In all neonates, we found highest abundance of *Streptococcus* and high frequencies of *Staphylococcus*, *Gemella* and *Rothia* after birth, which is in accordance with a previous study in elderly neonates (2 months old) without CLP [53]. Similarly, it was shown that neonates with CLP (3 days and 1-2 weeks old) presented relatively high abundance of the families *Streptococcaceae* and *Gemellaceae* [18] and high levels of *Staphylococcus* [*S*.] *aureus*, *S. epidermidis* and *Gemella haemolysans* [21]. However, the limitation of all those studies [18,21,52,53] is the missing comparison between CLP and control neonates. Regarding oral niches, we aimed to investigate two different niches (tongue and cheek) from the two expected metaniches (saliva-tongue-hard palate ‘S-T-HP’ and cheek-sublingual region ‘C-U’), since young adults showed three distinct microbial metaniches (plaque-gingival crevicular fluid ‘P-GCF’, ‘S-T-HP’ and ‘C-U’) [19] and ‘P-GCF’ will not be detectable in neonates without teeth. Metaniche ‘S-T-HP’ was characterized by significantly higher alpha diversity compared to ‘C-U’ in young adults, which can be explained by the presence of major salivary glands and high saliva flow rate found in the cheek [19]. Further, significant differences regarding beta diversity were found between ‘S-T-HP’ and ‘C-U’, e.g. significantly higher levels of *Streptococcus salivarius*, *Fusobacterium (pseudo)periodonticum*, *Prevotella melanogenica*, *Granulicatella adiacens* and *Veillonella dispar* in ‘S-T-HP’ and significantly higher levels of *Haemophilus parainfluenza* and *Gemella haemolysans* in ‘C-U’ [19]. Hence, similar differences regarding alpha and beta diversity between the oral niche tongue (T) and cheek (C) were expected in neonates. However, in contrast to adults, no microbial clustering into metaniches and no differences with regard to beta diversity were found in neonates between the oral niche tongue and cheek. Accordingly, Dominguez-Bello *et al* [52]. found no differences between body niches (skin, oral, nasopharyngeal, gut) after birth regarding beta diversity and assumed that the human microbiota is homogeneously distributed across the body in its initial maturation process. Similarly, we assume that differences in beta diversity with regard to oral niches develop later in life during the oral microbiome development (as found in young adults [19]. Since no differences were found between the groups (CLP vs. CTRL, CLo vs. CPo vs. UCLP vs. BCLP, high vs. low cleft severity) with respect to sampling niche or with combined niches regarding beta diversity, we decided to solely present data with ignored sampling site for reduction of figure count and better readability. No bias resulted by ignoring the sampling site in the presentation of beta diversity since individual analyses of each sampled niche did not lead to different results. While alpha diversity was not significantly different between both niches, we found some differences with tongue samples presenting significantly lower alpha diversity in CLP neonates compared to control neonates at both time points, while those differences were not seen in cheek samples. These differences might be explained by the higher overall alpha diversity found in tongue samples (metaniche ‘S-T-HP’) compared to cheek samples (metaniche ‘C-U’) in healthy adults [19]. Hence, alpha diversity might increase earlier during the maturation process in tongue samples compared to cheek samples and this physiological increase might be altered by clefting.

Regarding postnatal microbiome maturation within the first weeks of life, our results presented a significant increase of alpha diversity in both groups. This is in accordance with a rising alpha diversity found in the oral microbiome from 6 to 24 months after birth [54] as well as during the first three years of life [53,55]. Within the first weeks of life, we detected an overall of 39 genera (117 OTUs) in neonates at two time points (3 days after birth, 4–5 weeks after birth), which is partly in accordance with a previous study detecting a total of 356 OTUs in children at 6 time points (1.9, 7.7, 13.2, 19.7, 39.0 and 48.6 months-of-age) [53]. Considering the first time point (2 months of age), the authors detected 31 OTUs in the saliva using next generation sequencing [53], which is lower compared to our results. Differences might be explainable by methodology used by Dashper *et al* [53]. choosing a different sampling method (pipetting of saliva) and another variable region (the V4 region) of the bacterial 16S rRNA genes. Instead, we used tongue smear samples and the V1-V3 region for NGS. Notably, in young adults with oral health 300 OTUs were found [19] and, overall, 775 oral bacterial taxa were detected so far [14] indicating that the number of bacterial species raises with age. Moreover, we found a proportional increase of *anaerobic species* from T0 to T1 in both groups. In the control group, representing ‘natural maturation’ of oral microbiota, an increase of *stress-tolerant species* as well as an aggravation of *Haemophilus*, *Veillonella*, *Bergeyella*, *Lactobacillus*, *Actinomyces*, *Atopobium* and *Pseudomonas* was observed. Similarly, *Haemophilus parainfluenzae* presented high abundance in 2-month-old neonates without CLP [53].

Notably, the presence of orofacial clefts resulted in significant differences regarding composition and complexity of the oral microbiota compared to control neonates. Alpha diversity was significantly lower in CLP neonates compared to controls and lowest alpha diversity was found in neonates with high cleft severity. Interestingly, CLP neonates showed significantly lower microbial complexity of tongue samples compared to control tongue samples. Similarly, lower alpha diversity was also found in saliva samples from non-syndromic complete CLP (Ø 17 months) compared to controls [20] and saliva samples were shown to have a similar microbial composition as tongue and hard palate samples in healthy adults (metaniche saliva-tonge-hard palate ‘S-T-HP’) [19]. The natural increase of alpha diversity [53–55] is disturbed by changes in the oral (micro-)environment due to clefting (and subsequent PSIO) and this developmental alteration increases with increasing cleft severity. Further, a higher abundance of *Pseudomonas* and *Gram-positive species* and lower abundance of *facultative anaerobic* and *Gram-negative species* was seen in CLP neonates.

With respect to orofacial clefts’ influence on oral microbiome maturation within the first weeks of life, a clustering into neonates with CLP and control neonates was seen based on a dendrogram and were supported by MDS and CCA analyses where the CLP group formed a distinct cluster at T1. CLP neonates showed significantly higher values of *Enterobacteriaceae (Citrobacter, Enterobacter, Escherichia-Shigella, Klebsiella), Enterococcus, Bifidobacterium, Corynebacterium, Lactocaseibacillus, Staphylococcus, Acinetobacter and Lawsonella* compared to controls. In accordance with our findings, oral samples from neonates with CLP presented an increase of species belonging to the genera *Klebsiella*, *Neisseria* and *Enterococcus* from 1–2 weeks until 8–18 weeks after birth [21]. Differences in oral microbiota were also seen in older CLP infants (17-months-old) compared to controls, e.g. lower amounts of *Lautropia* and *Bacillus* in CLP neonates [20]. The higher levels of *Staphylococcus* (a skin colonizer [56] found in CLP neonates might be explainable by the affection of extraoral structures leading to a mixture between skin and oral microbiota. Similarly, in neonates born via C-section (with more contact to mothers’ skin during birth), higher levels of Staphylococcus were acquired compared to vaginal birth [52]. Further, we found an elevation of *Gram-negative* and a decrease of *Gram-positive species* from T0 to T1. The most well-known biofilm in the oral cavity is dental plaque, a structurally and functionally organized complex microbial community [57] that contains several bacteria playing a major role in specific formation stages, e.g. *Streptococcus mutans* [58*], Veillonellae* [59*], Enterococcus faecalis and Streptococcus salivarius* [60*], Actinomyces sp., Tannerella forsythia, Fusobacterium nucleatum, Spirochaetes, Synergistetes*, *Lactobacillus sp., Streptococcus sp. and Candida albicans* [61]. It is known that a higher prevalence to plaque biofilm formation can be found in infants and adolescents with CLP [62]. Since neonates lack teeth, the increase of biofilm-forming species might rather play a role in the formation of ‘salivary pellicles’ that build a thin conditioning layer on soft tissues in the oral cavity [63–65], e.g. on the tongue and cheek located near major (cheek) and minor (tongue) salivary glands [66].

Considering CLP types, two patients with very low Severity Scores (CLo = 2, incomplete CPo = 4) clustered in the control group at T1 indicating that low cleft severity do not change the oral microbiome composition. Further, we found similarities between UCLP and BCLP neonates and differences between CPo and UCLP/BCLP neonates at both time points. Similarly, previous studies presented differences in microbiota composition between UCLP/BCLP and CPo neonates (1–5 months [21] and 6–7 months old [22]. At T0, CPo neonates showed higher levels of *anaerobic* and *facultative anaerobic species* compared to BCLP/UCLP neonates (high cleft severity as well of *Streptococcus* sp compared to UCLP. At T1, CPo neonates presented higher abundances of *Prevotella histicola* and *Staphylococcus* compared to both UCLP/BCLP, greater abundance of *Klebsiella pneumoniae*, *Streptococcus infantis* and sp., *Haemophilus* sp. *(e.g. H. parainfluenzae, H. haemolyticus)*, *Corynebacterium kroppenstedtii* and *Neisseria sicca* compared to BLCP as well as higher levels of *Campylobacter concisus*, *Prevotella melaninogenica*, *Pseudomonas marginalis* and *Veillonella atypica* compared to UCLP. Notably, *Muribaculaceae*, *Escherichia*, *Staphylococcus* and *Lachnospiraceae* are dominant genera in the neonatal nasal microbiome [67]. Moreover, BCLP/UCLP neonates (high cleft severity) presented significantly lower levels of *Gram-negative* and significantly higher levels of *Gram-positive* species at T0. Compared to controls, BCLP neonates presented higher numbers of *Lactobacillus gasseri* and *Bifidobacterium longum* compared to CPo at T1. Interestingly, the skin microbiota contains several beneficial species, e.g. *Lactobacillus* [68], an *oxygen-tolerant anaerobe*, which is also used as probiotic supplement to promote oral health [69,70]. Compared to CPo at T1, UCLP neonates presented significantly higher abundance of *Rothia mucilanginosa* and *Citrobacter freundii*, which was associated with infections and multidrug resistance [71]. Dissimilarities between CPo and UCLP/BCLP neonates can be explained by the different phenotypes: CPo are characterized by an insufficient separation of nasal and oral cavity, however, without affection of extraoral structures, while clefting in UCLP and BCLP affects extraoral and intraoral anatomical structures [2,4]. Therefore, the oral microbiota of both CPo and UCLP/BCLP neonates might be composed by oral and nasal microbiota components, while in UCLP and BCLP the insufficient lip closure might also lead to a mixture with skin microbiota. Further, different oxygen levels in the oral cavity in CPo compared to UCLP/BCLP might impact the growth of *anaerobic species* in CPo neonates. Interestingly, the CLo patient (LKG_018) clustered in the control group at T1. The microbiome of that patient was characterized by high levels of Gram-negative species and lower levels of Gram-positive species, which was a similar distribution compared to control neonates. These similarities between the CLo patient and controls can be explained by the mild manifestation of orofacial cleft with an incomplete affection of the lip and alveolus (LAHSHAL: la - - - - -) without involvement of intraoral structures. Notably, the CLo patient and two neonates with incomplete CPO (LKG-002 and LKG-004) received no treatment with palate plates (which was performed in most CLP neonates, see Table 1) and clustered with controls at T1. This might indicate that not only clefting but also cleft-associated treatments (palate plates) might alter oral microbiome maturation. Hence, not all neonates with orofacial clefts suffer from altered microbiota and a mild affection (without need for treatment with palate plates) might be most beneficial for ‘natural’ oral microbiome maturation.

The limitation of this study are differences between the CLP and control group with regard to weight, height, birth mode, antibiotic intake intrapartum (mothers with C-section) and different nutrition modes. Moreover, the CLP group showed significantly lower median weight at T0 (3,120 g at T0) compared to the control group with a median weight (3,480 g at T0) in conformity with the European average [72] (Table 2). Compatible with the lower birth weight at T0, the CLP group presented a slightly lower median height at T0 (51 cm) (Table 2). Previous studies detected an impact of birth mode [73] and intrapartum antibiotic therapy [18] on the oral microbiome composition. However, different birth modes as well as antibiotic intake intrapartum were distributed equally in both groups of our study and we detected no differences between vaginal and C-section born neonates in both groups. Notably, in most of our study participants a positive correlation between C-section an antibiotic intake intrapartum was observed, which is in accordance to standard protocols as prophylactic use of antibiotics 30 minutes prior to C-section was shown to prevent post-surgical infections [74]. Interestingly, a previous study investigating mothers’ and neonates’ microbiome according to delivery mode (vaginal vs. C-section) did not detect an impact of cephalosporin given to mothers several hours prior to C-section [52]. With regard to nutrition, the control group in our study was exclusively breast-fed, whereas the CLP group showed mixed nutrition modes that can’t be excluded due to the well-known feeding issues in CLP neonates after birth. To insure proper daily food intake, different nutrition methods like bottle feeding with Haberman feeder [12] and in severe cases supplementary use of nasogastric tube feeding [13] are necessary in neonates with CLP (Table 2). For statistical comparison, we evaluated different nutrition methods including information of the nutrition mode (breast-feeding, bottle-feeding and/or tube feeding) and nutrition (breast milk, partly breast party artificial food, completely artificial food). As expected, we detected significant differences with regard to nutrition between the CLP and control group, which is consistent with reported feeding difficulties in the CLP group [10,11]. However, different nutrition modes were distributed equally with regard to cleft phenotype and severity, e.g. breast-milk bottle-feeding was found in neonates with CPo (LKG-005) and UCLP neonates (LKG-007, LKG-011) and postnatal tube feeding was seen in CPo (LKG-002, LKG-016) and BCLP neonates (LKG-10, LKG-012) (Table 1). Subgroup analyses showed a trend towards higher levels of *facultative anaerobic species* in oral microbiota of breast milk (bottle) fed (breast or bottle-feeding) neonates compared to formula-bottle-fed (partially or complete artificial baby food) neonates (d.n.s.), which is in accordance to previous studies detecting higher abundance of *Lactobacillus* and *Bifidobacteriae* (*Gram-positive* and *facultative anaerobic species* [75,76] used as probiotics in preventive treatments [69,70,76] in the gut microbiome of 4-months old infants. Taken together, there might be a positive effect of breast-milk (bottle) feeding with regard to oral microbiome maturation, however, larger sample sizes are needed in future studies.

In conclusion, the present study revealed that the postnatal ‘core’ oral microbiome is characterized by an evenly distributed *Streptococcus* spp.-dominated microbial community with high abundance of *Staphylococcus*, *Gemella* and *Rothia* and that the ‘symbiotic’ early life oral maturation process is characterized by an increase of alpha diversity, an elevation of distinct genera (*Haemophilus*, *Veillonella*, *Bergeyella*, *Lactobacillus*, *Actinomyces*, *Atopobium*, *Pseudomonas*) and phenotypes (*anaerobe* and *stress-tolerant species*) associated with health. While distinct oral metaniches were not seen in neonates, there is some evidence, that the formation of metaniches starts after birth with an increased alpha diversity in tongue samples. This knowledge is necessary for a better understanding of the regular maturation process of oral microbiota as well as for an early detection of pathological developments. However, physiological maturation of the microbiota as exemplified by an increase of alpha diversity is disturbed by clefting and this developmental alteration increases with higher cleft severities. OFC impact early life oral microbiota development in terms of increased levels of *Enterobacteriaceae* (*Citrobacter*, *Enterobacter*, *Escherichia-Shigella, Klebsiella*), *Enterococcus*, *Staphylococcus* and *Acinetobacter*. Further, we could show that the involvement of intraoral and extraoral structures by clefting plays a major role presented by a mixture with skin microbiota in UCLP/BCLP neonates and a mixture with nasal microbiota in CPo neonates. The consequence of our results might arrogate a standard monitoring of oral microbiota in neonates with OFC (at least in case of higher severity) within the first year of life to identify an increase of potential pathogens, e.g. *Enterobacteriaceae*, and to define a dysbiosis panel. Future studies should also focus on the oral microbiome after surgical (lip and/or palate) cleft closure compared to controls to analyse whether cleft closure alone guides oral microbiome maturation towards a physiological, ‘symbiotic’ environment. Higher prevalence of plaque, gingivitis and periodontitis in children, adolescents and adults with orofacial clefts [62] and the correlation of higher levels of beta-hemolytic *streptococci* with increased risk for palatal dehiscence after surgical cleft closure [22] speak against this theory. Therefore, we suppose that dysbiosis developed within the first year of life might not be normalized by surgical interventions only. If cleft closure alone is not sufficient to normalize the oral microbiota development process, further studies are essential to investigate whether preventive strategies can help to reduce oral dysbiosis after birth and before surgical interventions, e.g. by postponing of surgery for 2–4 weeks in severe cases for therapeutic approaches.

## Supplementary Material

Supplemental Material

## Data Availability

Participants of the study will be informed about the findings of the trial, but only if desired. Raw sequencing data together with non-personal metadata is available through a publicly accessible database (BioProject: PRJNA881235). Upon reasonable request and after approval by data protection commissioner, further de-identified metadata may be made available by the corresponding author once the trial is completed. Raw sequencing data is available through the National Center for Biotechnology Information (NCBI) Sequence Read Archive (SRA) under BioProject PRJNA881235. https://www.ncbi.nlm.nih.gov/sra/PRJNA881235.

## Abbreviations

A: Alveolous (LAHSHAL code)
ABM: antibiotic intake mother intrapartum
BCLP: Bilateral cleft lip and cleft palate
c: caesarian
CCA: constrained correspondence analysis
CLo: Cleft lip only
CLP: Cleft lip and cleft palate
cm: centimeter
CPo: Cleft palate only
CTRL: control
d: days
d.n.s.: data not shown
f: female
g: grams
H: Hard Palate (LAHSHAL code)
HP: Hard Palate
L: Lip (LAHSHAL code)
m: male
MDS: multidimensional scaling
n: number
NAM: Nasoalveolar Molding
NGS: next generation sequencing
noABM: no antibiotic intake by mother intrapartum
OFC: Orofacial clefts
p: Cleft of the primary palate
pAM: Passive Alveolar Molding
ps: cleft of the primary and secondary palate
s: Cleft of the secondary palate
S: Soft Palate (LAHSHAL code)
T: Tongue
T0: Time Point T0 after birth
T1: Time point T1 4-5 weeks after birth
UCLP: Unilateral cleft lip and cleft palate
v: vaginal
